# Empirical estimation of Young’s modulus for biological tissue mimics using acoustic impedance measurements: A study on agar gel tissue phantoms

**DOI:** 10.1371/journal.pone.0320705

**Published:** 2025-04-14

**Authors:** Shinri Morodomi, Kazushi Ito, Satoru Maegawa, Yoshihiro Ujihara, Shukei Sugita, Masanori Nakamura

**Affiliations:** 1 Department of Electrical and Mechanical Engineering, Graduate School of Engineering, Nagoya Institute of Technology, Aichi, Japan; Gdańsk University of Technology, POLAND

## Abstract

The mechanical properties of biological tissues are significant biomarkers for diagnosing various diseases. The aim of this study was to develop an empirical formula to estimate Young’s modulus from the acoustic impedance measured by scanning acoustic microscopy. Agar, a material with mechanical properties similar to those of biological tissues, was prepared at concentrations ranging from 5% to 20%. The acoustic impedance was measured by scanning acoustic microscopy, and Young’s modulus was determined via indentation testing. The results showed that both the acoustic impedance and Young’s modulus increased with agar concentration. Theoretical models did not accurately describe the relationship between the acoustic impedance *Z* and Young’s modulus *E*; however, the empirical formula $E=9.2835\;\times \;10^{-6}Z^{2}\;-\;21.6347\;\times \;10^{6}$ (with *E* in Pa and *Z* in Ns/$m^{3}$) provided a better fit. This formula could potentially be used to estimate Young’s modulus for biological tissues, aiding in the realistic analysis of stress fields and understanding the etiology of various diseases.

## Introduction

The mechanical properties of biological tissues are important biomarkers of health and pathological conditions in various diseases [1]. Measuring the mechanical properties of biological tissues, such as their density and elasticity, has valuable diagnostic applications. For instance, Yeh et al. [2] reported liver stiffness increased with fibrosis severity and showed the potential clinical value of ultrasonic elasticity imaging for assessing fibrosis severity. Krouskop et al. [3] demonstrated that breast carcinomas, particularly infiltrating ductal carcinomas, were significantly stiffer than other tissues at high strain levels, and highlighted the potential of elastography to differentiate tissue types based on stiffness, with implications for diagnosing breast and prostate pathologies. Lin et al. [4] revealed that patients with later stages of chronic kidney disease had stiffer kidneys, and thus concluded that renal elasticity is associated with proteinuria and rapid renal deterioration in patients with chronic kidney disease.

Various experiments methods are used to determine mechanical properties of biological samples. The tensile test is the most common method [5]. In this test, both ends of a sample are gripped, and it is pulled until it breaks or reaches a defined strain. Although biological tissues are typically inhomogeneous, viscoelastic materials that exhibit nonlinear stress–strain behavior, a linear elastic model described by Young’s modulus and Poisson’s ratio is generally used for representing the overall mechanical characteristics of a sample of interest. A uniaxial tensile test is a well-established and standardized method, making it easy to perform and interpret. For heterogeneous materials with potential directional dependencies (anisotropy), the uniaxial tensile test can reveal variations in mechanical properties along different axes. It also provides apparent mechanical properties of the material, which are often sufficient to understand its macroscopic characteristics. A “biaxial tensile test” applies tension in two directions on the specimen at once, providing a more comprehensive understanding of the material’s behavior under complex loading conditions. One of the limitations of the biaxial testing lies in the need of specialized testing machines with precise control over loads and displacements in multiple directions. Furthermore, stress concentrations and boundary effects near grips can lead to non-uniform strain fields outside the central region and near the gripping part, complicating data interpretation [6,7]. Digital image correlation (DIC) is a non-contact optical technique that measures surface deformation by tracking image patterns, suitable for delicate or heterogeneous materials. While DIC is versatile and compatible with various mechanical tests, its accuracy depends on high-quality speckle patterns, lighting, and camera systems, limiting its use for relatively small-scale measurements [8]. Compression tests, which evaluate material behavior under compressive loads, are straightforward and adaptable but require precise specimen preparation to ensure accuracy [9]. Atomic force microscopy (AFM) has emerged as a method for quantifying mechanical properties at the nanoscale [10]. AFM can achieve a vertical subnanometer resolution and a lateral resolution of a few nanometers, but it requires a large amount of time to measure large surfaces. Because of this limitation, care must be taken to avoid sample dehydration during measurements that are not performed under liquid conditions. Moreover, the scanned area is relatively limited; output images have lengths on the order of 0.1 mm. Additionally, the probe must be changed for each measurement or sample to avoid contamination, making AFM costly. Parameter setting is difficult for inexperienced operators although some machines perform this function automatically. These difficulties make AFM unsuitable for evaluating the mechanical properties of biological tissues with a large surface area. Micropipette aspiration measures mechanical properties of small, soft materials like cells by applying suction pressure and analyzing deformation [11]. Although effective for studying individual cells, it is time-consuming and requires skilled operators for precise pipette handling and alignment. Other than these mechanical loading tests, some studies employ microfluidics-based techniques to characterize stiffness of samples [12,13]. Compared with traditional mechanical loading tests, the microfluidics-based techniques have high throughput, but are weak in the accurate quantification of mechanical properties. Furthermore, it is basically limited to small samples, exclusively to cells and difficult to apply for tissues.

Numerical analyses also play an important role in characterizing mechanical properties of materials. The finite element method (FEM) is a numerical technique used to solve complex problems in engineering and physics. FEM can model how materials deform under various loads (e.g., tension, compression, shear). The combination of FEM and inverse problems is particularly powerful for material characterization. Olson et al. developed computational techniques that use surface force measurements to create three-dimensional stiffness maps of breast tissue, aiming to refine and automate manual breast exams [14]. Odin et al. presented a method for determining the mechanical properties of mandibular bone using inverse analysis [15]. Moreover, Zhang et al. used the optimization method combined with FEM to inversely determine the mechanical properties of the iris in vivo based on [16].

Scanning acoustic microscopy (SAM) uses ultrasound signals to image the speed of sound in and the acoustic impedance of samples. The scanning area of SAM is on the order of several square millimeters. The acoustic impedance mode of SAM provides data on the local distribution of acoustic impedance in a cross section of the sample without requiring slicing. Owing to these advantages, SAM has been used to evaluate the acoustic properties of both soft and hard tissues. Saijo et al. [17] reported that the speed of sound in an atherosclerotic lesion is higher than that in unlesioned intima. Strohm and Kolios [18] scanned MCF7 breast cancer cells with SAM to characterize their acoustic properties. Hasegawa et al. [19] compared the acoustic velocity of biopsied iliac bones from normal and osteoporotic subjects and reported altered elasticity in the latter. Hatori et al. [20] applied SAM to dentistry to analyze the speed of sound in rat periodontal ligament at various developmental stages and demonstrated that it increases over the course of development. Although these studies demonstrate acoustic differences between normal and pathological tissues or cells, interpreting the corresponding differences in their mechanical properties remains challenging. If Young’s modulus could be derived from the acoustic impedance measured by SAM, it would enable the effective mapping of Young’s modulus across biological tissues using SAM. This approach would be especially advantageous for measuring relatively large sample areas.

The aim of the present study is therefore to establish an empirical formula that determines Young’s modulus from the acoustic impedance obtained by SAM. For this purpose, we measured the acoustic impedance and Young’s modulus for agar at different concentrations to characterize the relationship between them.

## Materials and methods

### Sample preparation

**Fig 1:**
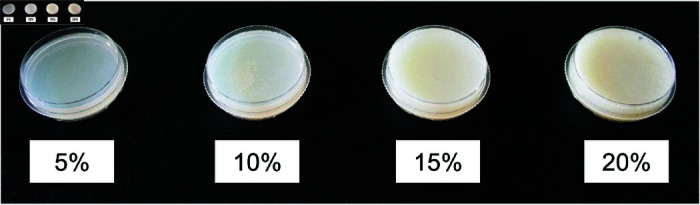
Agar samples.

Agar is a useful material for replicating the physical properties of human soft tissue because it has similar mechanical properties that can be adjusted based on concentration [21–24]. By adjusting the agar concentration, it is possible to create materials with various Young’s moduli in a range similar to that for biological tissues [25]. In the present study, agar samples were prepared at four different concentrations, ranging from 5% to 20% in 5% increments, relative to the weight of distilled water. Fig 1 exhibits agar samples at these concentrations. Five samples were made for each concentration. Distilled water (10 mL) produced by an ultrapure water production system (Direct-Q UV 3, Yamato Scientific Co., Ltd.) was vacuum degassed for more than an hour using a vacuum degassing machine (VD, AS ONE Corporation, DA-20D, ULVAC KIKO, Inc.). Agar powder (01059-85, Nacalai Tesque, Inc.) was added to the vacuum-degassed distilled water, heated, and dissolved. The dissolved agar was poured into a 35 mm dish (150460, Thermo Fischer Scientific Inc.) and refrigerated until it solidified. The dish was treated with a hydrophilic treatment by irradiating it with an excimer lamp for 15 min.

### Measurement of acoustic impedance

**Fig 2:**
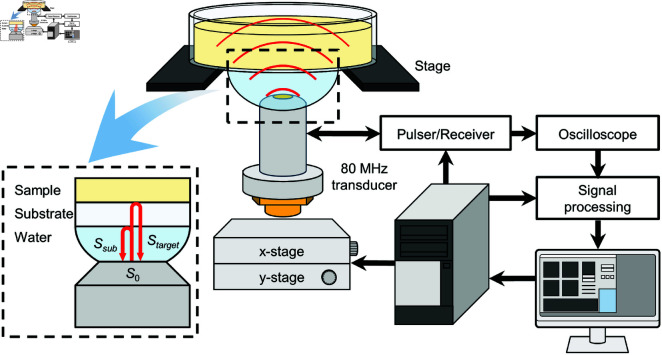
Schematic of SAM system.

The acoustic impedance of the agar was measured with a scanning acoustic microscope (AMS-50AI, Honda Electronics Co., Ltd.). Fig 2 shows a schematic of the experimental setup. The agar sample to be measured was placed on the stage above a transducer with a center frequency of 80 MHz (HTD80-2025, Honda Electronics Co., Ltd.), which radiates an acoustic signal and receives the reflected signal. Distilled water was used as the coupling fluid between the dish and the transducer. The sample was scanned in the *x*- and *y*-directions with aid of a mechanical stage to obtain the acoustic impedance distribution of the sample. The measurement area was 4.8 mm × 4.8 mm, and scans were obtained with a resolution of 300  ×  300 pixels in this area.

To calculate the acoustic impedance of the agar, the acoustic impedance $Z_{sub}$ of the substrate and that $Z_{ref}$ of a reference material with a known acoustic impedance needed to be set. $Z_{sub}$ was set to 2.37 MNs/$m^{3}$ to match that of the material of the dish bottom (polystyrene) [26].

Distilled water was used as the reference material to set $Z_{ref}$. The sound speed *c* and density *ρ* for the distilled water were estimated from the water temperature measured with a thermometer (3003XT101B, BOMATA) based on existing literature [27–30]. The acoustic impedance $Z_{ref}$ of the reference material was then determined from


$$\begin{array}{ccc} Z=\rho c, & & \end{array}$$ (1)

where *Z* is equal to $Z_{ref}$.

The acoustic impedance *Z* of the agar samples was measured after the samples had been stored at room temperature for a sufficient time. Measurements were conducted four times for each sample. The acoustic impedance in the measurement area was averaged to obtain a representative value for each measurement.

### Measurement of Young’s modulus

**Fig 3:**
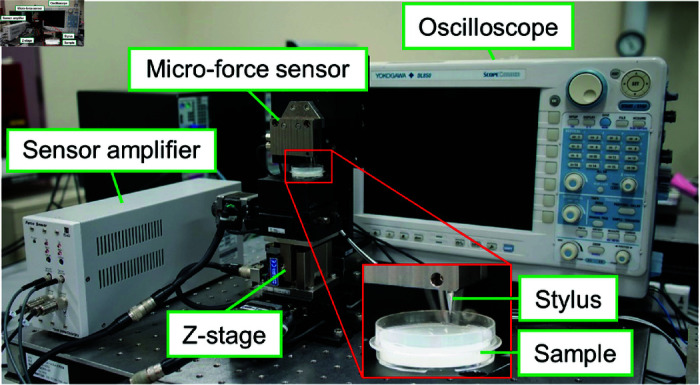
Indentation tester.

**Fig 4:**
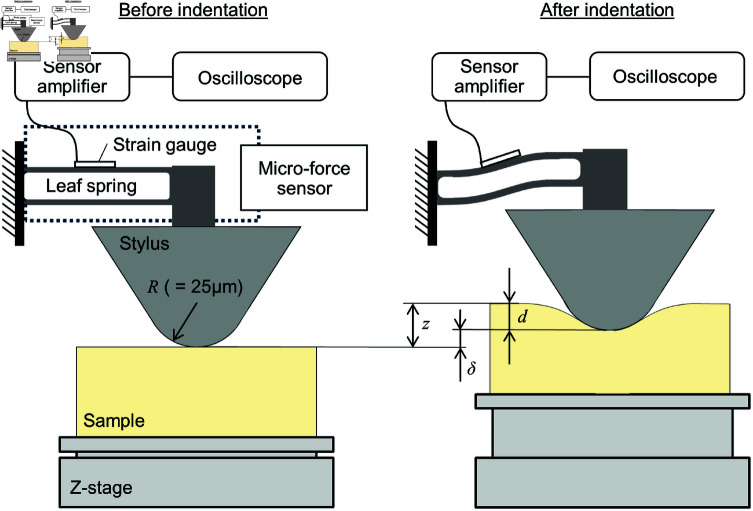
Schematic of indentation tester system.

Young’s modulus for the agar sample was measured with a custom-made indentation tester. Figs 3 and 4 present a photo and a schematic of the indentation tester, respectively. The indentation tester consists of a micro-force sensor (THK Precision Co., Ltd), a stylus (DM45505, Tokyo Seimitsu Co., Ltd.) fixed to the sensor and a Z-stage (KHE06008-CH, Suruga Seiki Co., Ltd.). The stylus is supported by a leaf spring (THK Precision Co., Ltd) installed inside the sensor. When a force is applied to the stylus, the leaf spring bends, causing the stylus to move vertically.

The bridge voltage signal from the strain gauge attached to the leaf spring is amplified by a sensor amplifier (FSA202S, THK Precision Co., Ltd.), and the amplified voltage *V* is recorded with an oscilloscope (DL850, Yokogawa Electric Co., Ltd.).

The magnitude of the received force *F* was calculated from the recorded voltage *V* as


$$\begin{array}{ccc} F=\lambda V, & & \end{array}$$ (2)

where *λ* is a coefficient that was provided by the company that manufactured the strain gauge (THK Precision Co., Ltd.). Given the force *F*, the deflection of the spring *δ* can then be obtained as


$$\begin{array}{ccc} \delta =\frac{F}{k}, & & \end{array}$$ (3)

where *k* is the stiffness of the spring. Finally, the indentation depth *d* for the stylus is calculated as


$$\begin{array}{ccc} d=z-\delta & & \end{array}$$ (4)

In the experiments, the specimen placed on the stage was raised by the Z-stage controlled via a stepping motor (PK523HPB-C17, Oriental Motor Co., Ltd.) at a rate of 10 µm/s such that the specimen was quasi-statically indented with the stylus. The parameter values used were $\lambda =2.635\;\times \;10^{-2}$ N/V and $k=5.86\;\times \;10^{-3}$ N/µm, provided by a manufacturer of the micro-force sensor (THK Precision Co., Ltd).

Each sample was indented at 20 different locations using a stylus with a tip radius *R* of 25 µm. Young’s modulus *E* was determined from the relationship between the fitting force *F* and the indentation depth *d* using Hertz’s contact theory described as


$$\begin{array}{ccc} F=\frac{4}{3}\frac{E}{\left(1-\nu^{2}\right)}R^{\frac{1}{2}}d^{\frac{3}{2}}, & & \end{array}$$ (5)

where *ν* is Poisson’s ratio. (Eq 5) was fit to the measurement data for an indentation depth range of 0–3 µm using MATLAB R2023a (9.14.0.2254940, MathWorks). Poisson’s ratio *ν* for the agar sample was assumed to be 0.499, indicating its incompressibility.

### Relationship between acoustic impedance and Young’s modulus

Theoretically, Young’s modulus *E* is described as a function of the acoustic impedance *Z* as follows. According to the wave equation, the sound speed *c* is given by


$$\begin{array}{ccc} c=\sqrt{\frac{K}{\rho }}, & & \end{array}$$ (6)

where *K* is the bulk modulus. If the sample is an isotropic linear material, *K* can be expressed as


$$\begin{array}{ccc} K=\frac{E}{3(1-2\nu )} & & \end{array}$$ (7)

using Young’s modulus *E* and Poisson’s ratio *ν*. Thus, combining (Eq 1), (6), and (7) yields the relationship between the acoustic impedance and Young’s modulus as


$$\begin{array}{ccc} E=\frac{3(1-2\nu )}{\rho }Z^{2}=\alpha Z^{2}, & & \end{array}$$ (8)

where *α* is the coefficient of $Z^{2}$ to be determined by fitting (Eq 8) to the experimental data.

In the present study, we also used the empirical formula


$$\begin{array}{ccc} E=\alpha Z^{2}+\beta & & \end{array}$$ (9)

to express the relationship between the acoustic impedance and the Young’s modulus, because (Eq 8) did not accurately represent the data, as shown in the following section. Here, *α* and *β* are constants to be determined by fitting.

## Results

**Fig 5:**
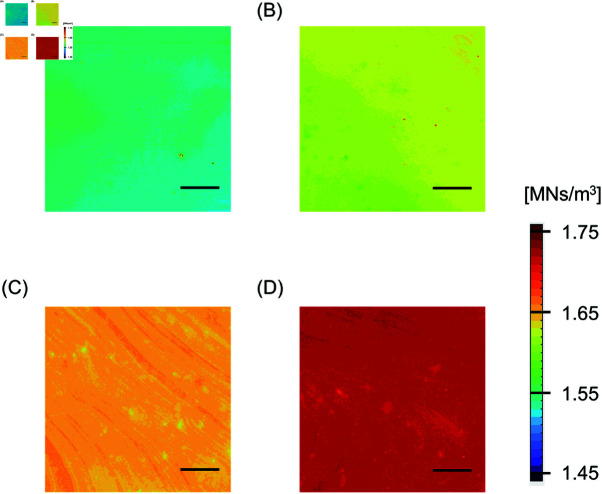
Representative acoustic impedance color-coded maps for agar samples with concentrations. (A) 5%, (B) 10%, (C) 15%, and (D) 20%. Bars: 1 mm.

**Fig 6:**
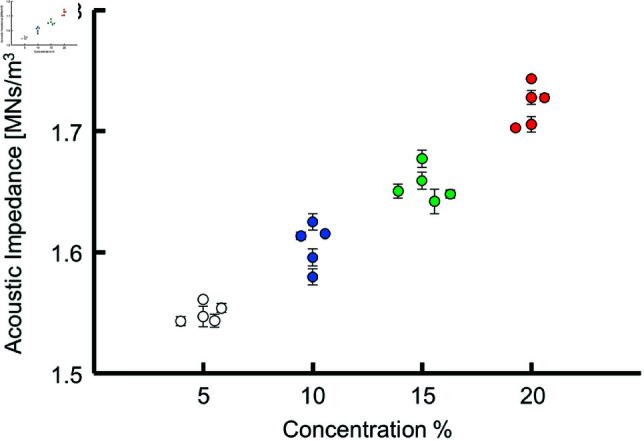
Acoustic impedance of each agar sample plotted against its concentration.

Fig 5 shows color-coded acoustic impedance images for agar samples prepared with different concentrations. The acoustic impedance of the agar appeared mostly uniform within the measurement area. As the agar concentration was increased from 5% to 20%, the acoustic impedance of the sample increased. Fig 6 shows the average acoustic impedance of each sample plotted against its concentration. Table 1 presents mean ± SD of the acoustic impedance of the samples. The acoustic impedance increased monotonically with agar concentration.

**Table 1:** Acoustic impedance of the agar samples (mean ± SD) [MNs/$m^{3}$]

5%	10%	15%	20%
1.547 ± 0.009	1.580 ± 0.007	1.642 ± 0.010	1.706 ± 0.007
1.543 ± 0.005	1.596 ± 0.007	1.677 ± 0.007	1.743 ± 0.002
1.543 ± 0.004	1.625 ± 0.007	1.660 ± 0.007	1.728 ± 0.006
1.569 ± 0.002	1.615 ± 0.001	1.648 ± 0.003	1.703 ± 0.000
1.554 ± 0.004	1.613 ± 0.003	1.651 ± 0.006	1.728 ± 0.003

**Fig 7:**
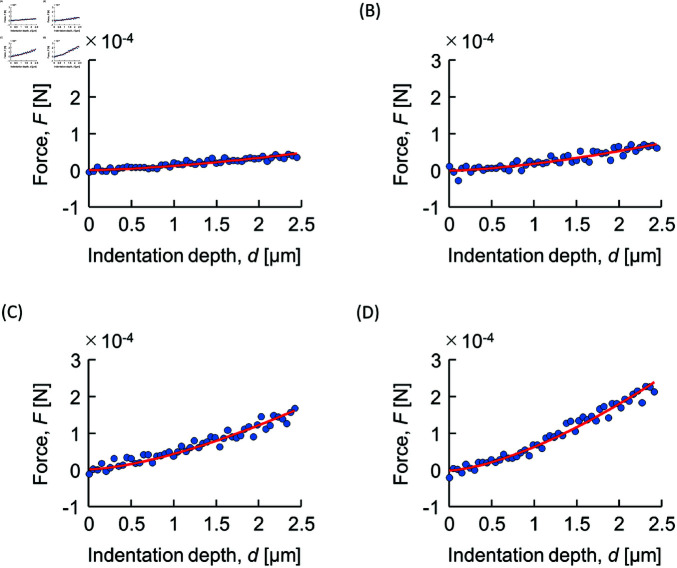
Representative plots of force against indentation depth for agar concentrations. (A) 5%, (B) 10%, (C) 15% and (D) 20%. Lines were obtained by fitting () to the data.

**Fig 8:**
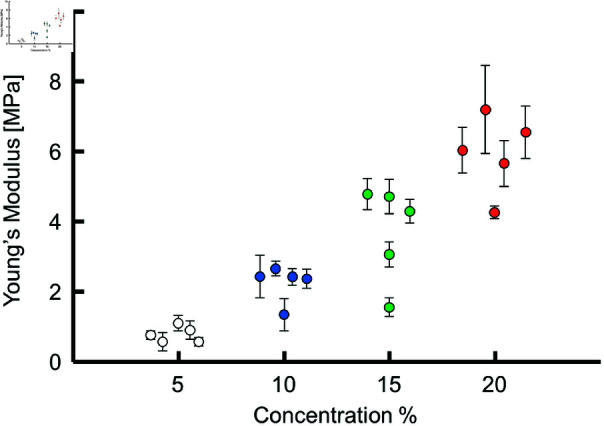
Young’s modulus for each agar sample plotted against its concentration.

Fig 7 shows representative force–indentation depth plots. As the agar concentration was increased, the reaction force against indentation by the stylus increased. The red lines in Fig 7 show the results of fitting using (Eq 5) to the force-indentation depth data, demonstrating a good fit. Fig 8 shows Young’s modulus obtained by fitting (Eq 5) to the force-indentation depth data for each sample, which is seen to increase monotonically with agar concentration. Table 2 presents mean ± SD of the Young’s modulus of the samples in numerical form.

**Table 2:** Young’s modulus of the agar samples (mean ± SD) [MPa]

5%	10%	15%	20%
0.760 ± 0.125	1.341 ± 0.459	1.552 ± 0.268	4.261 ± 0.180
1.102 ± 0.216	2.418 ± 0.231	4.709 ± 0.492	7.194 ± 1.251
0.898 ± 0.262	2.430 ± 0.599	4.290 ± 0.338	5.658 ± 0.653
0.569 ± 0.117	2.364 ± 0.274	3.060 ± 0.353	6.037 ± 0.654
0.570 ± 0.261	2.653 ± 0.208	4.782 ± 0.448	6.547 ± 0.749

**Fig 9:**
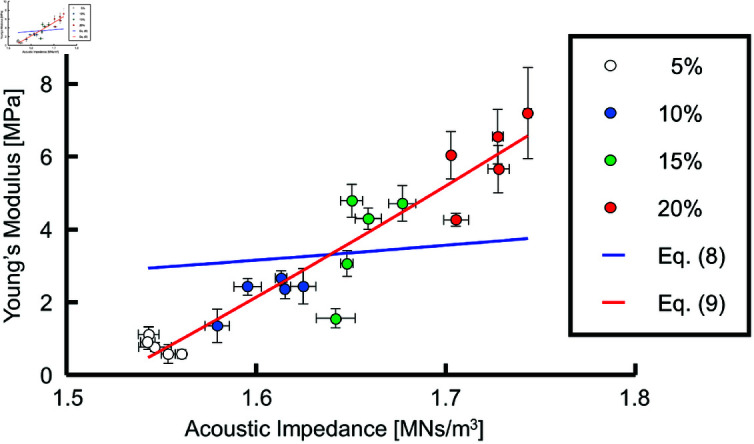
Young’s modulus plotted against acoustic impedance, both averaged over each sample. Blue line: obtained by fitting (Eq 8), $E=1.2336\times 10^{-6}Z^{2}$. Red line: obtained by fitting (Eq 9), $E=9.2835\times 10^{-6}Z^{2}-21.6347\times 10^{6}$.

Fig 9 shows Young’s modulus plotted against the acoustic impedance, with both quantities averaged for each sample. A least-squares regression analysis demonstrated that (Eq 8) (blue line) did not account well for the experimental data (*R*^2^ = 0.22). In contrast, (Eq 9) (red line), with an added bias term, accurately represented the measurement data ($R^{2}$ = 0.90). The obtained equation was


$$\begin{array}{ccc} E=9.2835\times 10^{-6}Z^{2}-21.6347\times 10^{6}, & & \end{array}$$ (10)

where the units of *E* and *Z* are Pa and Ns/$m^{3}$, respectively.

## Discussion

In this study, we measured the acoustic impedance and Young’s modulus for agar at concentrations ranging from 5% to 20% and examined the relationship between them. The fitting results using (Eq 8) showed that it did not accurately describe the measured data. (Eq 9), however, yielded a good fit. The use of (Eq 9) would enable Young’s modulus to be calculated from the acoustic impedance measured by SAM.

Although there is a theoretical basis for (Eq 8), which describes a quadratic relationship between the acoustic impedance and Young’s modulus, the introduction of a bias term in (Eq 9) was necessary to obtain a good empirical model for our data. In the present study, the estimated bias term *β* was  − 21.6347 MPa; such a negative intercept is unrealistic if we take into account that Young’s modulus for a material with zero acoustic impedance would equal *β*. At present, it remains unclear why adding this term to the theoretically described equation was necessary to obtain a good fit. Although (Eq 9) aligns with the theory in a sense that the Young’s modulus increases quadratically with acoustic impedance, it is important to bear in mind that (Eq 9) is an empirical equation that has only been demonstrated for an acoustic impedance range of 1.54-1.74 MNs/$m^{3}$.

According to (Eq 8), Poisson’s ratio for agar can be estimated from the coefficient *α* under the assumption that the density *ρ* of agar is 1000 kg/$m^{3}$. As seen in Supporting Information (S1 Fig, S1 Table), the density of agar samples was approximately 1000 kg/$m^{3}$ and did not vary significantly across the 5% to 20% concentration range (one-way ANOVA, *p* = 0.79). Extending this relationship to Eq (9) yields a Poisson’s ratio of 0.498, which agrees with the assumed value in the indentation test. This indicates that the empirical formula obtained is valid in terms of the value of Poisson’s ratio.

The acoustic impedance measured in this study ranged from 1.54 to 1.74 MNs/$m^{3}$, and Young’s modulus from 0.57 to 7.19 MPa. The acoustic impedance of aneurysmal aortic regions has been reported to range from 1.48 to 1.65 MNs/$m^{3}$ [31]. Young’s modulus for elastin and smooth muscle cells, the major components of blood vessels, is generally in the range of 0.1-2.0 MPa [32]. The measured ranges of the acoustic impedance and Young’s modulus in this study approximately encompass those for vascular tissues, suggesting the potential to calculate the Young’s modulus distribution of vascular tissues using the derived equation.

In this study, the acoustic impedance and Young’s modulus of agar were measured at different locations. As the acoustic wave emanated from the bottom of the dish, the measured acoustic impedance was for the bottom of the sample. In contrast, Young’s modulus was measured with the indentation tester at the top surface of the sample. For confirmation, we measured Young’s modulus for one agar sample at both the top and bottom surfaces and found no significant difference between the two. Therefore, it is considered acceptable to correlate the acoustic impedance at the bottom surface of the agar with Young’s modulus measured at the top surface.

In looking at Fig 6 and Fig 8, we found that the Young’s modulus of agar exhibited greater variation compared to the acoustic impedance, which can be attributed to two primary reasons. Firstly, the error associated with contact determination in the indentation test plays a significant role. The Young’s modulus was calculated using Hertz’s contact theory applied to the force-indentation curve, starting from a manually determined contact point. This manual selection introduces variation in the calculated Young’s modulus, whereas the acoustic impedance measurement using SAM does not involve such manual steps, resulting in more consistent acoustic impedance values. Secondly, the effect of surface drying of the agar during the indentation test contributes to the variation. The upper surface of agar exposed to air is prone to drying, making it brittle and potentially leading to variations in the Young’s modulus or causing the indenter tip to penetrate the brittle surface. In contrast, the SAM-based acoustic impedance measurement is performed on the bottom surface of the agar in the dish, which is not exposed to air, thereby minimizing the drying effect. These factors collectively explain why the Young’s modulus of agar shows greater variation compared to the acoustic impedance.

We used a transducer with a center frequency of 80 MHz. The spatial resolution of SAM is dependent on the center frequency, and an 80 MHz transducer provides a spatial resolution of approximately 20 µm [33]. Higher-frequency transducers provide a finer spatial resolution. Reportedly, a 320 MHz transducer would allow the measurement of the acoustic impedance of intracellular components [34]. However, to evaluate the mechanical properties at the vascular tissue scale, the transducer used in this study would be sufficient.

Studies using finite element analysis to determine the stress field within the blood vessel walls often define uniform material properties for the vascular model [35,36]. However, real blood vessels are composed of multiple materials such as smooth muscle cells, elastin, and collagen, each with a different Young’s modulus, and thereby show spatially heterogeneous mechanical characteristics [37]. Some studies proposed a hypothesis that abnormal stresses within vascular tissues trigger the onset and progression of vascular diseases [38,39]. Functionally graded materials (FGMs), which exhibit spatially varying properties, offer a more realistic approach by mimicking the natural heterogeneity of vascular tissues. Research on FGMs, such as stress analysis in functionally graded thick-walled cylinders [40–42], demonstrates their potential to improve stress distribution modeling in vascular walls. Integrating FGM models with advanced techniques like scanning acoustic microscopy (SAM) can provide detailed maps of local mechanical properties, enabling more precise stress analysis and insights into vascular disease mechanisms. This approach holds promise for advancing the diagnosis and treatment of vascular pathologies by better capturing the mechanical complexity of blood vessels.

Previous studies investigating Young’s modulus for agar [25,43,44] have reported values up to concentrations of approximately 5%. Li et al. [25] measured Young’s modulus for agar at concentrations from 0.5% to 5% using a laser-generated surface acoustic wave phase velocity dispersion technique and reported that it increases quadratically with concentration. Young’s modulus for 5% agar obtained in the present study (0.57-1.1 MPa) was similar to the value of 1.3 MPa reported by Li et al. [25]. Extrapolation of their results, however, implies that Young’s modulus for 20% agar is approximately 27 MPa, equivalent to that for electrospun tubular scaffolds [45]. The measurements in the present study demonstrated that Young’s modulus for an agar concentration of 20% was approximately 7 MPa, about a quarter of what would be estimated by extrapolating the results of Li et al. [25]. Such a discrepancy suggests that while Young’s modulus for agar increases quadratically with concentrations up to 5%, this trend does not continue when the concentration is extended to 20%.

Agar with a concentration above 5% is considered brittle [46]. It is difficult to measure Young’s modulus for brittle materials using mechanical loading tests such as tensile and compression tests because the samples are easy to break. The SAM-based method proposed in this study can measure Young’s modulus without applying any external force, providing a way to characterize the mechanical properties of relatively brittle materials such as mineralized tissues.

The stress-strain relationship in soft tissues is typically characterized by nonlinear behavior. However, it remains poorly understood how such material-specific nonlinear mechanical properties influence acoustic impedance. As an initial step toward elucidating how the mechanical properties of biological soft tissues are reflected in acoustic impedance, this study employed a simplified linear elastic model to investigate the relationship between acoustic impedance and Young’s modulus at zero strain. While the assumption of linear elasticity may not fully capture the mechanical complexity of soft tissues, it remains a widely utilized approach in finite element analyses of biological materials [35,36]. Although the stress and strain values derived under this assumption may lack quantitative accuracy, they provide a sufficient basis for qualitative interpretation of the underlying phenomena. The applicability of the empirical formula derived in this study under conditions of applied strain remains uncertain. Future work will focus on examining the relationship between acoustic impedance and Young’s modulus in agar under varying strain conditions to further assess the potential utility of ultrasonic microscopy in characterizing soft tissue mechanics.

## Conclusion

In this study, an empirical formula was successfully derived to estimate Young’s modulus from the acoustic impedance measured by SAM, demonstrating a high correlation ($R^{2}$ = 0.90) and encompassing ranges typical of vascular tissues. This represents a significant step forward in non-invasive mechanical characterization, offering a practical and efficient alternative to conventional methods like AFM. The formula enables the analysis of brittle materials such as high-concentration agar, which are challenging to test with traditional mechanical approaches, and aligns with reported values for biological tissue mimics. However, the model’s reliance on an empirical bias term raises questions about its theoretical underpinnings, and its applicability is currently limited to homogeneous agar samples and a specific acoustic impedance range (1.54-1.74 MNs/$m^{3}$). Additionally, while the resolution provided by the 80 MHz transducer is sufficient for vascular tissue analysis, it may not be suitable for finer biological features. Future studies should focus on addressing these theoretical and material-specific limitations, extending the model’s applicability to heterogeneous tissues, and validating it across diverse biological contexts.

## Supporting information

S1 Fig Density for each agar sample plotted against its concentration. Statistical analysis was conducted with one-way analysis of variance (ANOVA). No significant difference was found in the density of samples across the 5% to 20% concentration range (*p* = 0.79).
(TIF)

S1 TableDensity for each agar sample plotted against its concentration.Density of agar samples, *ρ* [kg/$m^{3}$](PDF)

## References

[1] Cho DH, Aguayo S, Cartagena-Rivera AX. Atomic force microscopy-mediated mechanobiological profiling of complex human tissues. Biomaterials. 2023;303:122389. doi: 10.1016/j.biomaterials.2023.122389 37988897 PMC10842832

[2] Yeh WC, Li PC, Jeng YM, Hsu HC, Kuo PL, Li ML, et al. Elastic modulus measurements of human liver and correlation with pathology. Ultrasound Med Biol 2002;28(4):467–74. doi: 10.1016/s0301-5629(02)00489-1 12049960

[3] Krouskop TA, Wheeler TM, Kallel F, Garra BS, Hall T. Elastic moduli of breast and prostate tissues under compression. Ultrason Imaging 1998;20(4):260–74. doi: 10.1177/016173469802000403 10197347

[4] Lin HY, Lee YL, Lin KD, Chiu YW, Shin SJ, Hwang SJ, et al. Association of renal elasticity and renal function progression in patients with chronic kidney disease evaluated by real-time ultrasound elastography. Sci Rep. 2017;7:43303. doi: 10.1038/srep43303 28240304 PMC5327389

[5] Scholze M, Safavi S, Li KC, Ondruschka B, Werner M, Zwirner J, et al. Standardized tensile testing of soft tissue using a 3D printed clamping system. Hardware X. 2020;8:e00159. doi: 10.1016/j.ohx.2020.e00159 35498242 PMC9041186

[6] Sugita S, Matsumoto T. Novel biaxial tensile test for studying aortic failure phenomena at a microscopic level. Biomed Eng Online. 2013;12:3. doi: 10.1186/1475-925X-12-3 23305508 PMC3560224

[7] Sugita S, Matsumoto T, Ohashi T, Kumagai K, Akimoto H, Tabayashi K, et al. Evaluation of rupture properties of thoracic aortic aneurysms in a pressure-imposed test for rupture risk estimation. Cardiovasc Eng Tech 2012;3(1):41–51. doi: 10.1007/s13239-011-0067-1

[8] Reu PL, Sweatt W, Miller T, Fleming D. Camera system resolution and its influence on digital image correlation. Exp Mech. 2015;55:9–25. doi: 10.1007/s11340-014-9886-y

[9] Navindaran K, Kang JS, Moon K. Techniques for characterizing mechanical properties of soft tissues. J Mech Behav Biomed Mater. 2023;138:105575. doi: 10.1016/j.jmbbm.2022.105575 36470112

[10] Krieg M, Fläschner G, Alsteens D, Gaub BM, Roos WH, Wuite GJL, et al. Atomic force microscopy-based mechanobiology. Nat Rev Phys. 2019;1:41–57. doi: 10.1038/s42254-018-0001-7

[11] González-Bermúdez B, Guinea GV, Plaza GR. Advances in micropipette aspiration: applications in cell biomechanics, models, and extended studies. Biophys J 2019;116(4):587–94. doi: 10.1016/j.bpj.2019.01.004 30683304 PMC6383002

[12] An L, Ji F, Zhao E, Liu Y, Liu Y. Measuring cell deformation by microfluidics. Front Bioeng Biotechnol. 2023;11:1214544. doi: 10.3389/fbioe.2023 37434754 PMC10331473

[13] Nakamura M, Ono D, Sugita S. Mechanophenotyping of B16 melanoma cell variants for the assessment of the efficacy of (-)-epigallocatechin gallate treatment using a tapered microfluidic device. Micromachines (Basel) 2019;10(3):207. doi: 10.3390/mi10030207 30934576 PMC6470883

[14] Olson LG, Throne RD, Rusnak EI, Gannon JP. Force-based stiffness mapping for early detection of breast cancer. Inverse Prob Sci Eng 2021;29(12):2239–73. doi: 10.1080/17415977.2021.1912036

[15] Odin G, Savoldelli C, Bouchard PO, Tillier Y. Determination of Young’s modulus of mandibular bone using inverse analysis. Med Eng Phys 2010;32(6):630–7. doi: 10.1016/j.medengphy.2010.03.009 20466581

[16] Zhang K, Qian X, Mei X, Liu Z. An inverse method to determine the mechanical properties of the iris in vivo. Biomed Eng Online. 2014;13:66. doi: 10.1186/1475-925X-13-66 24886660 PMC4047431

[17] Saijo Y, Ohashi T, Sasaki H, Sato M, Jorgensen CS, Nitta S. Application of scanning acoustic microscopy for assessing stress distribution in atherosclerotic plaque. Ann Biomed Eng 2001;29(12):1048–53. doi: 10.1114/1.1424912 11853254

[18] Strohm EM, Kolios MC. Measuring the mechanical properties of cells using acoustic microscopy. Annu Int Conf IEEE Eng Med Biol Soc. 2009;6042-5. doi: 10.1109/IEMBS.2009.5334535 19964888

[19] Hasegawa K, Turner CH, Recker RR, Wu E, Burr DB. Elastic properties of osteoporotic bone measured by scanning acoustic microscopy. Bone 1995;16(1):85–90. doi: 10.1016/s8756-3282(94)00013-1 7742089

[20] Hatori K, Saijo Y, Hagiwara Y, Naganuma Y, Igari K, Iikubo M, et al. Acoustic diagnosis device for dentistry. In: Sasaki K, Suzuki O, Takahashi N, editors. Interface Oral Health Science 2016. Singapore: Springer; 2017, pp. 181–201. doi: 10.1007/978-981-10-1560-1_16

[21] Normand V, Lootens DL, Amici E, Plucknett KP, Aymard P. New insight into agarose gel mechanical properties. Biomacromolecules 2000;1(4):730–8. doi: 10.1021/bm005583j 11710204

[22] Ebenstein DM, Pruitt LA. Nanoindentation of soft hydrated materials for application to vascular tissues. J Biomed Mater Res A 2004;69(2):222–32. doi: 10.1002/jbm.a.20096 15057995

[23] Madsen EL, Hobson MA, Shi H, Varghese T, Frank GR. Tissue-mimicking agar/gelatin materials for use in heterogeneous elastography phantoms. Phys Med Biol 2005;50(23):5597–618. doi: 10.1088/0031-9155/50/23/013 16306655 PMC3769983

[24] Dahmani J, Laporte C, Pereira D, Belanger P, Petit Y. Predictive model for designing soft-tissue mimicking ultrasound phantoms with adjustable elasticity. IEEE Trans Ultrason Ferroelectr Freq Control 2020;67(4):715–26. doi: 10.1109/TUFFC.2019.2953190 31725375

[25] Li C, Huang Z, Wang RK. Elastic properties of soft tissue-mimicking phantoms assessed by combined use of laser ultrasonics and low coherence interferometry. Opt Express. 2011;19(11):10153–-63. doi: 10.1364/OE.19.010153 21643273

[26] Tanoren B. Scanning acoustic microscopy in examining cerebral aneurysm. Appl Acoust. 2021;179:109052. doi: 10.1016/j.apacoust.2021.108052

[27] Bigg PH. Density of water in SI units over the range 0–40∘C. Br J Appl Phys. 1967;18:1659. doi: 10.1088/0508-3443/18/4/315

[28] Jones FE, Harris GL. ITS-90 density of water formulation for volumetric standards calibration. J Res Natl Inst Stand Technol. 1992;97(3):335–-40. doi: 10.6028/jres.097.013 28053436 PMC4909168

[29] Greenspan M, Tschiegg CE. Speed of sound in water by a direct method. J Res Nat Bur Stand 1957;59(4):249. doi: 10.6028/JRES.059.028

[30] Lubbers J, Graaff R. A simple and accurate formula for the sound velocity in water. Ultrasound Med Biol. 1998;24(7):1065–-8. doi: 10.1016/s0301-5629(98)00091-x 9809641

[31] Tanoren B, Parlatan U, Parlak M, Kecoglu I, Unlu MB, Oztas DM, et al. Aortic aneurysm evaluation by scanning acoustic microscopy and Raman spectroscopy. Anal Methods 2021;13(39):4683–90. doi: 10.1039/d1ay01133b 34549754

[32] Herman IP. Physics of the Human Body. Springer; 2016: p. 548. doi: 10.1007/978-3-319-23932-3

[33] Bilen B, Gokbulut B, Kafa U, Heves E, Inci MN, Unlu MB. Scanning acoustic microscopy and time-resolved fluorescence spectroscopy for characterization of atherosclerotic plaques. Sci Rep 2018;8(1):14378. doi: 10.1038/s41598-018-32788-2 30258115 PMC6158264

[34] Soon TTK, Sasaki R, Prastika EB, Kawaguchi Y, Kobayashi K, Hozumi N, et al. Evaluation of elastic change during the mitotic phase of murine breast cancer cells using scanning acoustic microscopy. Jpn J Appl Phys. 2022;61:SG1070. doi: 10.35848/1347-4065/ac54f7

[35] Qi L, Zhu W, Qian W, Xu L, He Y, Zhao F. The performance of a spherical-tip catheter for stent post-dilation: finite element analysis and experiments. Front Physiol. 2021;12:734565. doi: 10.3389/fphys.2021.734565 34531765 PMC8438231

[36] Nan J, Rezaei M, Mazhar R, Jaber F, Musharavati F, Zalnezhad E, et al. Finite element analysis of the mechanism of traumatic aortic rupture (TAR). Comput Math Methods Med. 2020;2020:6718495. doi: 10.1155/2020/6718495 32724330 PMC7364233

[37] Sugita S, Matsumoto T. Heterogeneity of deformation of aortic wall at the microscopic level: contribution of heterogeneous distribution of collagen fibers in the wall. Biomed Mater Eng 2013;23(6):447–61. doi: 10.3233/BME-130771 24165548

[38] Kataoka H, Yagi T, Ikedo T, Imai H, Kawamura K, Yoshida K, et al. Hemodynamic and histopathological changes in the early phase of the development of an intracranial aneurysm. Neurol Med Chir (Tokyo). 2020;60(7):319-–28. doi: 10.2176/nmc.st.2020-0072 32536660 PMC7358784

[39] Raaz U, Zöllner AM, Schellinger IN, Toh R, Nakagami F, Brandt M, et al. Segmental aortic stiffening contributes to experimental abdominal aortic aneurysm development. Circulation. 2015;131(20):1783-–95. doi: 10.1161/CIRCULATIONAHA.114.012377 25904646 PMC4439288

[40] Wang ZW, Zhang Q, Xia LZ, Wu JT, Liu PQ. Thermomechanical analysis of pressure vessels with functionally graded material coating. J Press Vessels Technol. 2016;138:011205. doi: 10.1115/1.4031030

[41] Salimi Bani M, Asgharzadeh Shirazi H, Ayatollahi MR, Asnafi A. A new model for the artificial aorta blood vessels using double-sided radial functionally graded biomaterials. Med Biol Eng Comput 2017;55(5):859–71. doi: 10.1007/s11517-016-1569-7 27629551

[42] Abdalla HMA, Casagrande D, De Bona F. A dynamic programming setting for functionally graded thick-walled cylinders. Materials (Basel) 2020;13(18):3988. doi: 10.3390/ma13183988 32916876 PMC7560176

[43] Manickam K, Machireddy RR, Seshadri S. Study of ultrasound stiffness imaging methods using tissue mimicking phantoms. Ultrasonics. 2014;54(2):621-–31. doi: 10.1016/j.ultras.2013.08.018 24083832

[44] Nayar VT, Weiland JD, Nelson CS, Hodge AM. Elastic and viscoelastic characterization of agar. J Mech Behav Biomed Mater. 2012;7:60–-68. doi: 10.1016/j.jmbbm.2011.05.027 22340685

[45] Thomas V, Donahoe T, Nyairo E, Dean DR, Vohra YK. Electrospinning of Biosyn(®)-based tubular conduits: structural, morphological, and mechanical characterizations. Acta Biomater 2011;7(5):2070–9. doi: 10.1016/j.actbio.2011.01.008 21232639

[46] Poudrel AS, Bouffandeau A, Demeet OL, Rosi G, Nguyen VH, Haiat G. Characterization of the concentration of agar-based soft tissue mimicking phantoms by impact analysis. J Mech Behav Biomed Mater. 2024;152:106465. doi: 10.1016/j.jmbbm.2024.106465 38377641

