# Supplementary figures and images for: Empirical estimation of Young’s modulus for biological tissue mimics using acoustic impedance measurements: A study on agar gel tissue phantoms
Source: PLoS One. 2025 Apr 14;20(4):e0320705. doi: 10.1371/journal.pone.0320705 (PMC11996209; doi:10.1371/journal.pone.0320705)

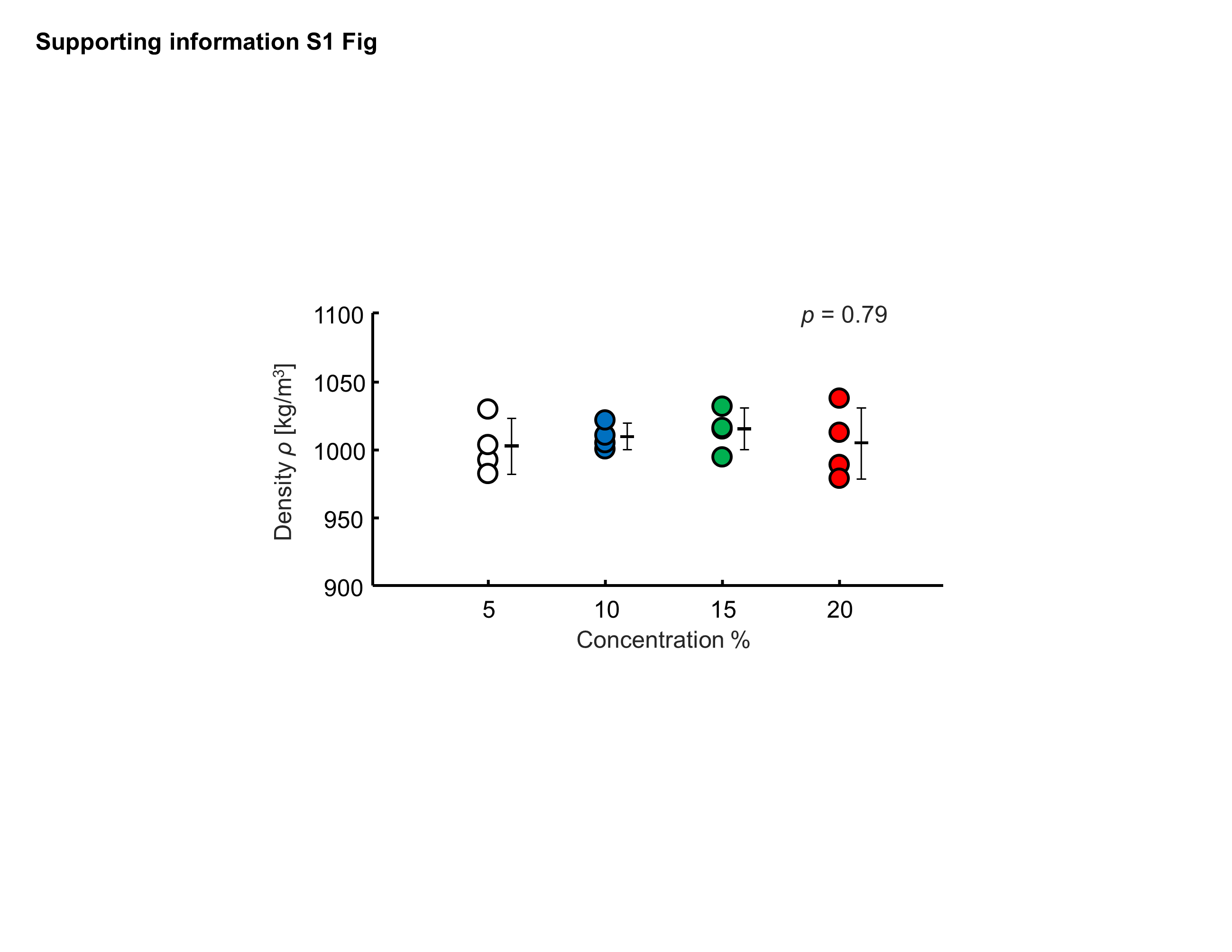

Supplement: S1 Fig — Statistical analysis was conducted with one-way analysis of variance (ANOVA). No significant difference was found in the density of samples across the 5% to 20% concentration range (p = 0.79). (TIF) [file pone.0320705.s001.tif]
