# Supplementary material for: Empirical estimation of Young’s modulus for biological tissue mimics using acoustic impedance measurements: A study on agar gel tissue phantoms
Source: PLoS One. 2025 Apr 14;20(4):e0320705. doi: 10.1371/journal.pone.0320705 (PMC11996209; doi:10.1371/journal.pone.0320705)
Supplement: S1 Table — Density of agar samples, ρ [kg/m3] (PDF) [file pone.0320705.s002.pdf]

S1 Table. Density of agar samples, ρ [kg/m^3]

5%	10%	15%	20%
993.3	1000.7	1015.4	1014.1
982.9	1005.8	996.0	1038.1
1030.4	1011.4	1033.3	989.9
1005.0	1022.2	1017.1	979.2
